# Long‐Term Prognosis of Teeth With Class II Furcation Involvement: A Retrospective Cohort Study

**DOI:** 10.1111/jcpe.14186

**Published:** 2025-06-02

**Authors:** P. Eickholz, T. Cordis, B. Dannewitz, B. Pretzl, M. Schröder, N. Lingwal, N. El Sayed

**Affiliations:** ^1^ Department of Periodontology, Center for Dentistry and Oral Medicine (Carolinum) Goethe‐University Frankfurt am Main Frankfurt am Main Germany; ^2^ Private Dental Practice Weilburg Germany; ^3^ Akademie für Zahnärztliche Fortbildung Karlsruhe Germany; ^4^ Center for Dentistry and Oral Medicine (Carolinum) Goethe‐University Frankfurt am Main Frankfurt am Main Germany; ^5^ Institute of Biostatistics and Mathematical Modeling Goethe University Frankfurt Frankfurt am Main Germany

**Keywords:** furcation involvement degree II, periodontal treatment, periodontitis stage III and IV

## Abstract

**Objective:**

To evaluate the survival of teeth with class II furcation involvement (FI) ≥ 5 years after active periodontal treatment (APT) and to identify the prognostic factors.

**Methods:**

All charts of patients having undergone APT at the Department of Periodontology of Goethe‐University Frankfurt, Germany, were screened for teeth with class II FI. APT had to be accomplished ≥ 5 years ago. Charts were analysed for data of class II FI teeth at baseline (T0), after APT (T1) and at the last supportive periodontal care (SPC/T2).

**Results:**

Two‐hundred and twenty‐two patients (age: 56.5 ± 10.1 years; 118 females; 35 active smokers; 17 diabetics, 154 stage III, 68 stage IV, 94 grade B, 128 grade C) presented 543 teeth with class II FI. Sixty‐one patients lost 93 teeth (17%), on average, over 108.4 ± 36.5 months of SPC. Logistic/Cox proportional hazards mixed‐model regressions associated increased tooth loss with irregular SPC (*p* = 0.023/0.073), premolar versus molar (*p* = 0.041/0.017), root canal filling (RCF) (*p* < 0.001) and multiple class II FI per tooth at T1 (*p* = 0.001/0.024).

**Conclusions:**

Of a total of 543 teeth with class II FI, 83% were retained for 108.6 ± 36.5 months. Multiple class II FI at T1, RCF, premolars and irregular SPC were found to compromise the long‐term prognosis of teeth with class II FI.

## 1 Introduction

Long‐term retention of natural teeth in a healthy, functional, aesthetically acceptable and painless state is the ultimate goal of dental/periodontal prevention and treatment (Hirschfeld and Wasserman 1978; Mombelli et al. 2014). Comprehensive periodontal treatment is quite effective: attachment and tooth loss (TL) occur rarely (Graetz et al. 2020). Many factors influencing the fate of periodontally compromised but treated teeth are already known. Patient‐related risk factors include age, interleukin‐1 polymorphism, smoking, irregular supportive periodontal care (SPC) (Eickholz et al. 2008; Pretzl et al. 2008; Muller et al. 2013; Lee et al. 2015) and individual biofilm control (Eickholz et al. 2008; Pretzl et al. 2008); tooth‐related risk factors include radiographic bone loss, furcation involvement (FI) (Dannewitz et al. 2006, 2016; Pretzl et al. 2008), probing pocket depth (PPD) and clinical attachment level (CAL) after active periodontal treatment (APT) (Petsos et al. 2021) and fixed and removable dentures (Muller et al. 2013; Petsos et al. 2021).

FI in multi‐rooted teeth still represents a major challenge for periodontal treatment. The furcation area is difficult, and in some cases impossible to access and clean. A registry‐based cohort study investigating 2,374,883 molars over 10 years reported loss rates for molars with FI class II of 21.9% and class III of 46.4% (Trullenque‐Eriksson et al. 2023). Class II FI in mandibular molars and buccal maxillary molars may be closed or transformed into class I FI (S. Jepsen, Gennai, et al. 2020) using regenerative treatment (Sanz et al. 2020). However, when considering treatment options for class II FI at maxillary interproximal sites (exclusive subgingival instrumentation [SI], open‐flap debridement [OFD], periodontal regeneration or resective techniques), the EFP S3 clinical guideline for treatment of stage I, II and III periodontitis failed to find significant advantages for any treatment (Dommisch et al. 2020) and made an open recommendation (Sanz et al. 2020). It was concluded that beyond class of FI, additional factors such as, for example, radiographic bone loss, residual pockets after treatment or the frequency of SPC may influence tooth survival (Dommisch et al. 2020). Thus, the Workshop recommended further research: for example, aggregation of raw data of existing studies to analyse the influence of factors other than class II and III FI and reporting the residual attachment of the remaining roots and the percentage of radiographically measurable bone loss. A retrospective analysis has already explored additional influencing factors for class III FI, identifying SI with adjunctive systemic antibiotics to favour retention of teeth with class III FI and baseline RBL and PPD at the start of SPC to deteriorate long‐term prognosis (Eickholz et al. 2021).

Thus, in this retrospective cohort study, we collected and analysed additional parameters other than the class of FI to explain the long‐term prognosis exclusively of teeth with class II FI.

## 2 Material and Methods

### 2.1 Patients

All charts of patients who had undergone APT since October 2004 at the Center for Dentistry and Oral Medicine of Goethe‐University Frankfurt/Main, Germany, were screened for the presence of teeth with class II FI. This study applies a protocol that we used for a recent study on long‐term survival of teeth with class III FI (Eickholz et al. 2021) to teeth with class II FI. The inclusion criteria are reported in detail there (Eickholz et al. 2021) and are provided in Supporting Information. Briefly, they are as follows:

– At least one tooth with class II FI at least on one furcation entrance prior to start of treatment. A tooth was classified as class II FI if at least one furcation entrance exhibited class II FI. Further, in accordance with the 2020 EFP S3 clinical guideline, we introduced the dichotomous variable ‘multiple class II FI’ per tooth (yes/no) (Sanz et al. 2020).

SI and SPC were provided in part by undergraduate dental students under the supervision of postgraduate dentists undergoing training for specialisation in periodontology of the German Society of Periodontology (DG PARO) (clinical courses of Goethe University Dental School) and certified specialists and in part by postgraduate dentists and certified specialists themselves. Periodontal surgery was always performed exclusively by postgraduate dentists and certified specialists for periodontology. The decision for surgical intervention was made in consultation with the Head of the Department (P.E.) or his deputy.

The trial was approved by the Institutional Review Board for Human Studies of the Medical Faculty of Goethe‐University Frankfurt/Main (174/19) and is registered at the German Register of Clinical Studies (Deutsches Register Klinischer Studien: DRKS) DRKS00028760.

### 2.2 Analysis of Patient Charts and Radiographs

The analysis of patient charts and radiographs has been reported in detail before (Eickholz et al. 2021) and is provided in the Supporting Information.

### 2.3 Statistical Analysis

The patient is defined as the statistical unit and the main outcome variable is TL during SPC (T1 to T2). The secondary outcome is transformation of FI class II to furcation closure or FI class I. All data were entered into an Excel data matrix (T.C.). Statistical analyses were performed using PC programs (Systat for Windows Version 13, Systat Inc., Evanston, IL, USA; IBM SPSS Statistics 24 software package, IBM, Chicago, IL, USA; R version 4.0.2) (Team 2021). For each individual, cigarette pack‐years were calculated. Taking into consideration that multiple class II FI at the same tooth may affect TL differently from a single class II FI, we created a binomial variable (multiple class II FI yes/no) (Sanz et al. 2020). Patient characteristics were described as absolute and relative frequencies (binomial and categorial variables) or means ± standard deviations (continuous variables). Variables were compared using univariate analysis (chi‐squared test for categorical variables and *t*‐test for continuous variables) between teeth retained and lost to identify possible independent variables for multivariate analysis. Univariate tests do not consider the clustered structure of the data. Change of FI between T0, T1 and T2 was categorised as improvement, stagnation or deterioration. Improvement is defined as transformation of FI class II to furcation closure or FI class I, and deterioration was defined as change into class III FI. Univariate chi‐squared tests were performed to compare change regarding treatment options, patient and tooth characteristics.

Using logistic mixed‐model regression and a mixed Cox proportional hazard model, patient‐related (regular SPC) and tooth‐related factors (multiple class II FI [yes/no], most severe PPD and CAL at T1, RCF, molar/premolar, SI) associated with TL could be identified. The patient was included as a random effect to consider the clustered structure of teeth within patients. Since this is an explorative study, *p*‐values are descriptive in nature.

## 3 Results

### 3.1 Patients

Two‐hundred and twenty‐two patients contributing 543 multi‐rooted teeth with class II FI were included in this analysis. During APT (T0–T1), 16 teeth (3%) with class II FI were extracted in 14 patients (6%). During SPC (T1–T2), 61 patients (27%) lost 93 teeth (17%) over a mean observation period of 108.6 ± 36.5 months. Ten teeth were lost exclusively due to caries, 9 exclusively due to endodontic, 51 exclusively due to periodontal and 1 exclusively due to prosthodontic (no reliable abutment tooth to support denture) reasons. Ten teeth were lost as a result of combined endodontic and periodontal reasons, one because of combined caries and periodontal reasons and one as a result of combined caries, endodontic and periodontal reasons. For 10 teeth, the indication for extraction could not be assessed.

The number of SPC visits ranged from 1 to 47. Table 1 provides the patient characteristics and Table 2 reports the respective diagnoses.

**TABLE 1:** Patient characteristics at T0.

Total number	222	
Observation time [months] (mean ± SD; M, LQ/UQ)	108.4 ± 36.5	103, 78/130
Females [*n* (%)]	118 (53)	
Age [years] (mean ± SD; M, LQ/UQ)	56.5 ± 10.1	57, 50/64
Diabetes [*n* (%)]	17 (8)	
Smoking		
Active smokers [*n* (%)]	35 (16)	
Non‐smokers [*n* (%)]	119 (53)	
Former smokers [*n* (%)]	68 (23)	
Pack years (mean ± SD; M, LQ/UQ)	11.1 ± 19.3	0, 0/17
Adjunctive systemic antibiotics [*n* (%)]	32 (14)	
Supportive periodontal care (SPC)		
Total number of SPC visits (mean ± SD; M, LQ/UQ)	16 ± 8.6	14, 10/19
Number of SPC visits per year (mean ± SD; M, LQ/UQ)	2.3 ± 0.8	1.7, 1.3/2
Regular SPC [*n* (%)]	58 (26)	

Abbreviations: SD: standard deviation; LQ: lower quartile; M: median; UQ: upper quartile.

**TABLE 2:** Diagnoses at T0: Periodontitis according to stage and grade [*n* (%)].

Stage	Extent	Grade B	Grade C	Total
III	Localised	32 (14)	21 (10)	53 (24)
III	Generalised	30 (14)	71 (32)	101 (46)
IV		32 (14)	36 (16)	68 (30)
Total		94 (42)	128 (58)	222

Univariate comparisons between patients losing teeth or not with regard to patient characteristics (sex, stage, grade, diabetes, smoking, regular/irregular SPC, systemic antibiotics adjunctive to SI, PCR, BOP) did not find any significant differences between the groups (Table 3).

**TABLE 3:** Patients with tooth loss (T1–T2) according to patient characteristics [*n* (%)].

	Retained	Lost	*p*
Total (219)	158 (72)	61 (28)	
Male (103)	72 (70)	31 (30)	0.485
Female (116)	86 (74)	30 (26)	
Localised stage III (54)	40 (74)	14 (26)	0.716
Generalised stage III (101)	74 (73)	27 (27)	0.732
Stage IV (65)	45 (69)	20 (31)	0.532
Grade B (93)	68 (73)	25 (27)	0.783
Grade C (126)	90 (71)	36 (29)	
Diabetes (17)	13 (76)	4 (24)	0.785
No diabetes (202)	145 (72)	57 (28)	
Current smoker (35)	24 (69)	11 (31)	0.607
Former/never smoker (184)	134 (73)	50 (27)	
Regular SPC (58)	46 (79)	12 (21)	0.156
Irregular SPC (161)	112 (70)	49 (30)	
Adjunctive systemic antibiotics (31)	24 (77)	7 (23)	0.480
No adjunctive systemic antibiotics (188)	134 (71)	54 (29)	
Plaque control record (T1)	34.4 ± 18.4	35.6 ± 17.4	0.643
Plaque control record (T2)	41.8 ± 20.0	45.6 ± 19.9	0.220
Bleeding on probing (T1)	13.9 ± 9.2	16.0 ± 8.7	0.122
Bleeding on probing (T2)	15.4 ± 9.3	17.6 ± 14.0	0.250

### 3.2 Teeth

A total of 28 maxillary first premolars and 515 molars with class II FI were included. The distribution in terms of molar type and jaw is given in Table 4.

**TABLE 4:** Distribution of teeth according to jaw and type [*n* (%)] at T0.

Total number		543		
Type	Premolar	First molar	Second molar	Third molar
Jaw	28 (5)	237 (44)	234 (43)	44 (8)
Maxilla [356 (66)]	28 (5)	158 (30)	156 (29)	14 (3)
Mandible [187 (34)]	0	79 (14)	78 (14)	30 (5)

Univariate comparisons between teeth retained and lost regarding tooth characteristics revealed significantly more loss of teeth with multiple than single class II FI at T0 and T1, RCF (yes/no), higher CAL and PPD at T1 (*p* < 0.001) (Table 5). From T0 to T1, 181 teeth (33%) exhibited transformation from FI class II to furcation closure or FI class I, 19 (4%) progressed to class III FI, 327 (60%) remained stable and 16 were extracted. Multi‐site class II FI transformed significantly more often (7%) from class II to III than single site (3%) (*p* = 0.002). From T1 to T2, 105 teeth (24%) improved, 85 (20%) deteriorated and 243 (56%) remained stable. Multi‐site class II FI did not deteriorate significantly more often (25%) than single site (18%) (*p* = 0.064). From T0 to T2, 204 teeth (38%) exhibited transformation of FI class II to furcation closure or FI class I, 55 (10%) progressed to class III FI, 174 (32%) remained stable and 109 were lost. Multi‐site class II FI deteriorated significantly more often (22%) than single site (11%) (*p* = 0.034).

**TABLE 5:** Tooth loss (T1–T2) according to tooth characteristics [*n* (%)].

	Retained	Lost	*p*
Total (527)	434 (82)	93 (18)	
Single class II FI (baseline/T0) (420)	359 (85)	61 (15)	< 0.001
Multiple class II FI (baseline/T0) (107)	75 (70)	32 (30)	
Single class II FI (start of SPC/T1) (442)	385 (87)	57 (13)	< 0.001
Multiple class II FI (start of SCP/T1) (65)	37 (57)	28 (43)	
Maxilla (345)	285 (83)	60 (17)	0.832
Mandible (182)	149 (82)	33 (18)	
Premolars (28)	24 (86)	4 (14)	0.632
First molars (235)	200 (85)	35 (15)	0.137
Second molars (223)	179 (80)	44 (20)	0.282
Crown (196)	154 (79)	42 (21)	0.080
No crown (331)	280 (85)	51 (15)	
Root canal filling (52)	33 (63)	19 (37)	< 0.001
No root canal filling (475)	401 (84)	74 (16)	
Vertical bone loss (45)	37 (82)	8 (18)	0.981
Horizontal bone loss (434)	358 (82)	85 (18)	
Radiographic bone loss at baseline (%)	40.4 ± 15.6	42.6 ± 16.3	0.238
Clinical attachment loss (baseline/T0)	6.7 ± 2.0	7.2 ± 2.2	0.041
Clinical attachment loss (start of SPC/T1)	5.5 ± 1.9	6.4 ± 2.4	< 0.001
Probing pocket depth (baseline/T0)	6.2 ± 1.8	6.7 ± 2.0	0.020
Probing pocket depth (start of SPC/T1)	4.6 ± 1.5	5.5 ± 2.0	< 0.001

### 3.3 Treatment

Of a total of 543 teeth with class II FI, 19 (4%) received oral hygiene instructions, risk factor modification and SPC exclusively. Four hundred teeth received exclusively step 1 treatment, SI and SPC. A further 144 teeth received additional step 3 treatment (Table 6a). Univariate comparisons between teeth retained and lost by treatment type revealed exclusive SI to be associated with less TL (*p* = 0.001), whereas access flap was associated with more TL (*p* = 0.002) (Table 6a). Resective furcation surgery is provided in Table 6b.

**TABLE 6a:** Tooth loss (T1–T2) according to treatment [*n* (%)].

	Retained	Lost	*p*
Total (527)	434 (82)	93 (18)	
Exclusively step 1 treatment (19)	12 (63)	7 (37)	0.058
Subgingival instrumentation (step 2) only (398)	340 (85)	58 (15)	0.001
Periodontitis therapy steps 2 and 3			
Open flap debridement only (OFD) (72)	50 (69)	22 (31)	0.002
Tunnel (3)	2 (67)	1 (33)	0.442
Regenerative treatment (23)	20 (87)	3 (13)	0.781
Resective furcation surgery (12)^a^	10 (83)	2 (17)	1.000

^a^ Two maxillary molars with trisection of both buccal roots.

**TABLE 6b:** Roots resected in resective treatment [*n* (%)].

	Retained	Lost	*p*
Total teeth (12)	10 (83)	2 (17)	1.000
Root amputation (6)	5 (83)	1 (17)	1.000
Mesio‐buccal root (2)	2 (100)	0	1.000
Disto‐buccal root (3)	2 (67)	1 (33)	0.455
Palatal root (1)	1 (100)	0	1.000
Trisection (4) (3: both buccal roots removed; 1: mesio‐buccal and palatal root removed)	3 (75)	1 (25)	0.529
Hemisection (2) (distal root removed)	2 (100)	0	0.520

Logistic mixed‐model regression identified regular SPC (*p* = 0.023) as well as molar versus premolar (*p* = 0.041) with less and RCF (*p* < 0.001) as well as multiple class II FI per tooth at T1 (*p* = 0.001) with more tooth loss (Table 7a). Mixed Cox proportional hazard model associated molar versus premolar (*p* = 0.017) and SRP (*p* = 0.039) with less TL and RCF (*p* < 0.001), maximum CAL (*p* = 0.001) and multiple class II FI per tooth at T1 (*p* = 0.024) with more TL (Table 7b).

**TABLE 7a:** Logistic mixed‐model regression to explain tooth loss (*n* = 527).

	Odds ratios	Lower confidence level	Upper confidence level	*p*
Regular SPC	0.267	0.086	0.832	0.023
Subgingival Instrumentation	0.404	0.123	1.332	0.137
Molar versus premolar	0.442	0.202	0.968	0.041
Maximum PPD (T1)	1.350	0.730	2.500	0.338
Maximum CAL (T1)	1.250	0.650	2.405	0.504
Root canal filling	8.107	2.490	26.399	< 0.001
Multiple class II FI (T0)	0.913	0.308	2.710	0.870
Multiple class II FI (T1)	9.027	2.415	33.743	0.001

Abbreviations: SPC: supportive periodontal care; PPD: probing pocket depth; CAL: clinical attachment level; FI: furcation involvement.

**TABLE 7b:** Cox proportional hazards mixed‐model regression to explain tooth loss (*n* = 527).

	Hazard ratios	Lower confidence level	Upper confidence level	*p*
Regular SPC	0.470	1.229	2.921	0.073
Subgingival Instrumentation	0.453	1.238	2.612	0.039
Molar versus premolar	0.538	1.382	2.447	0.017
Maximum PPD (T1)	0.739	1.658	2.942	0.117
Maximum CAL (T1)	2.113	3.844	27.535	0.001
Root canal filling	4.101	10.007	1484.634	< 0.001
Multiple class II FI (T0)	0.970	1.515	9.637	0.944
Multiple class II FI (T1)	2.491	3.091	244.306	0.039

Abbreviations: SPC: supportive periodontal care; PPD: probing pocket depth; CAL: clinical attachment level; FI: furcation involvement.

## 4 Discussion

The objective of this retrospective cohort study was to evaluate the survival of teeth with class II FI at least 5 years after APT and to identify the prognostic factors. All charts of patients who had undergone APT at the Department of Periodontology of Goethe‐University Frankfurt, Germany, since October 2004 were screened for teeth with class II FI prior to APT (T0). Charts were analysed for data of these teeth at T0, at the completion of APT and at the last SPC. Further, baseline radiographic bone loss at T0 and treatment were assessed. Two hundred and twenty‐two patients (154 stage III, 68 stage IV, 94 grade B, 128 grade C) contributed 543 teeth with class II FI. Sixty‐one patients lost 93 teeth (17%) over a mean observation period (SPC: T1–T2) of 108.4 ± 36.5 months. Logistic/Cox proportional hazards mixed‐model regressions associated irregular SPC (*p* = 0.023/0.073), premolar versus molar (*p* = 0.041/0.017), RCF (*p* < 0.001) and multiple class II FI per tooth at T1 (*p* = 0.001/0.024) with increased TL.

Molars with class II and III FI carry a substantially higher risk for TL than class I (Salvi et al. 2014; Graetz et al. 2015; Dannewitz et al. 2016). Numerous randomised controlled trials (RCTs) have compared OFD and regenerative treatment in class II FI in mandibular molars and buccal maxillary molars, providing solid evidence for closure or transformation into class I FI (S. Jepsen, Gennai, et al. 2020). Regenerative treatment is therefore recommended for this condition (Sanz et al. 2020). However, when looking at other treatment options for class II FI (e.g., exclusive SI, OFD, resective techniques), the 2019 European Workshop in Periodontology did not find any RCTs. Thus, the respective structured review (SR) had to rely on cohort studies. Six‐hundred and sixty‐seven patients contributed 2021 teeth with 1428 class II FI, 546 class III FI and 47 teeth where FI was not distinguished (Svardstrom and Wennstrom 2000). Data were very heterogeneous regarding the follow‐up and distribution of FI. A total of 1146 teeth with class II (80.3%) and 336 with class III (61.5%) FI survived 4–30.8 years after therapy (Dommisch et al. 2020). TL rates for molars with class II FI varied between 14% and 56% (both resective furcation surgery) (Dommisch et al. 2020; Dannewitz et al. 2016). The SR failed to identify significant advantages for any treatment (Dommisch et al. 2020) and issued open recommendations (Sanz et al. 2020). It was concluded that beyond the FI class, additional factors such as, for example, RBL and PPD at the start of/after treatment or SPC may influence tooth survival (Dommisch et al. 2020; Eickholz et al. 2021).

This study focused on teeth with class II FI, as a recent SR reported superior survival outcomes for class II than class III FI following periodontal treatment (Dommisch et al. 2020). Consequently, the class of FI was excluded as a differentiating factor in this analysis. Interestingly, it makes a big difference regarding TL whether a tooth exhibits single (13%) or multiple (43%) class II FI at T1. This may be in part due to class III FI that was underscored as multiple class II FI at T1 (Eickholz and Walter 2018). In a similarly designed study, class III FI at T1 resulted in 37% TL after 109 ± 33.5 months of SPC (Eickholz et al. 2021). However, our results demonstrate that actual multi‐site class II FI at T0 is more likely than single‐site class II FI to progress to class III FI at T1, increasing the risk for extraction during SPC. This study confirms to some extent the assumption that adherence to SPC facilitates tooth survival. Regular SPC is an established factor associated with improved tooth retention in general (Lee et al. 2015). However, whereas logistic multiple regression does identify regular SPC to be associated with tooth retention (*p* = 0.023), Cox proportional hazards mixed‐model regression does not (*p* = 0.073). Although residual pockets at the start of SPC are a well‐established predictor for TL in general (Matuliene et al. 2008; Petsos et al. 2021) for class II FI this association was not confirmed. Cox hazard multiple regression identified severest CAL per tooth to be associated with TL, whereas logistic regression did not. In cases of severe periodontitis, adjunctive use of systemic antibiotics has been shown to reduce residual pockets and further attachment loss despite treatment (Harks et al. 2015; Eickholz et al. 2019; Feres et al. 2012; Herrera et al. 2020; Benz et al. 2023). Although systemic antibiotics adjunctive to SI were associated with an improvement in the survival rate of teeth with class III FI (Eickholz et al. 2021), this is not confirmed for teeth with class II FI. Additionally, systemic antibiotics previously failed to reduce FI compared to placebo (Eickholz et al. 2016). In both logistic and Cox hazard multiple regression, RCF at T0 was clearly associated with greater TL in teeth with class II FI. Endodontic treatment/filling has previously been shown to be associated with increased risk of TL in molars (Pretzl et al. 2016; Dannewitz et al. 2016). Additional treatment (i.e., endodontic) results in an additional risk of complication. Further, RCFs are associated with an increased risk of vertical fractures, which regularly result in extraction (Langer et al. 1981). The use of posts for restoration of root‐canal‐filled teeth is associated with higher failure rates compared to restorations without posts (Willershausen et al. 2005). Resective treatment of FI regularly requires an RCF (S. Jepsen et al. 2021). Thus, resective FI treatment encompasses the complications that are inherent with RCFs if the root canal filling did not exist for other reasons (pulp necrosis) prior to resective treatment. A novel approach aims to preserve pulp vitality within the retained root by using metal trioxide aggregate, potentially avoiding or reducing these complications (K. Jepsen, Dommisch, et al. 2020; Tahmooressi et al. 2016; Ciardo et al. 2024). Ten‐year survival of molars with class II FI had been reported to be significantly different regarding subclasses of vertical attachment/bone loss (attachment/bone loss extending to the coronal third of the root: subclass A; middle third of the root: subclass B; apical third of the root: subclass C) with survival of 91% for subclass A, 67% for subclass B and 23% for subclass C (Tonetti et al. 2017). This study observes more severe CAL at T1 in lost than retained teeth with class II FI in univariate analysis. However, whereas Cox proportional hazards mixed‐model regression does identify maximum CAL per tooth to be associated with TL, logistic multiple regression does not. Further, neither bone loss relative to root length nor the type of bone loss (horizontal/vertical) in teeth with class II FI make any difference regarding TL. This discrepancy may be explained by the fact that Tonetti et al. investigated only patients adherent to SPC. However, in the present study, non‐adherence to SPC is a patient‐related factor determining instability in the logistic multiple regression model.

Over a mean observation period of 9 years, resective surgery resulted in 83% survival. More than 20 years ago, another group reported 93% survival 10 years after resective surgery of 123 molars with class II FI, 38 with class III, 12 with intrabony defects and 2 with endodontic lesions (Carnevale et al. 1998). The 93% survival was reported for a cohort investigated in a prospective clinical study including regular SPC. The study did not report patients' adherence to SPC. The actual retrospective study encompassed also patients not adhering to SPC. This may explain the different survival rates. Using univariate comparison, exclusive SI had a beneficial effect on the survival of teeth with class II FI, whereas exclusive step 1 treatment and OFD showed the contrary effect (Table 6a). In general, APT resulted in improvement of FI: 181 teeth (33%) showed transformation from class II FI to furcation closure or FI class I, 19 (4%) progressed to class III FI, 327 (60%) remained stable and 16 were extracted. During SPC, further improvement was observed: 105 teeth (24%) improved, 85 (20%) deteriorated and 243 (56%) remained stable. Overall from T0 to T2, 204 teeth (38%) exhibited transformation of FI class II to furcation closure or FI class I; only 55 (10%) progressed to class III FI, 174 (32%) remained stable and 109 were lost. This result was achieved in the vast majority of teeth exclusively with SI and SPC (i.e., repeated SI). Why were not more teeth with class II FI treated surgically? Beyond clinical parameters, both the strategic value of a respective tooth and the patient's willingness to undergo surgery significantly influence the treatment decision. For example, a second maxillary molar in a complete dentition of 28 teeth holds less importance for mastication compared to the same tooth adjacent to an edentulous space and intended to serve as an abutment tooth. In the first scenario, SI may be regarded as a less invasive ‘palliative treatment’ with a reasonable chance of retaining the tooth for some years (Dannewitz et al. 2006). If this tooth is finally lost, mastication is not compromised. In the second scenario, rehabilitation of masticatory function will rely on retention of the tooth, and both the dentist and the patient are more likely to consider a more invasive treatment. However, the parameter of strategic significance is difficult to account for in this type of analysis (Eickholz et al. 2021).

This study did not observe any association between sex, baseline diagnosis, diabetes, smoking, systemic antibiotics adjunctive to SI, plaque control or BOP at T0/T1 and retention of teeth with class II FI. In particular, factors with well‐documented impact on TL, such as diabetes, smoking and BOP index, failed to show an association in this study. However, diabetes, smoking and BOP index are patient‐related factors. This study investigated not TL per patient but only survival/loss of teeth with class II FI. Thus, it may be speculated that clinical parameters of the respective tooth have higher significance than patient‐related factors (Eickholz et al. 2008; Pretzl et al. 2008). Because of the fact that all patients by definition had to exhibit at least one tooth with class II FI, all patients were in at least localised stage III (complexity: class II FI) (Tonetti et al. 2018). Consequently, there was minimal difference in stage between patients. The assignment of grade, however, may have been influenced by bone loss at a tooth other than the one with class II FI. Thus, grade may be determined independently of the tooth with class II FI and may hold less relevance for its prognosis. In contrast to a similar cohort evaluating long‐term survival of teeth with class III FI, regular SPC was associated with a higher rate of survival in the present study (Eickholz et al. 2021).

What are the limitations of this analysis? First of all, this is a retrospective cohort study with a high risk of bias. Additionally, looking into regenerative surgery and the different types of resective furcation treatment, groups become quite small and analysis tends to be underpowered. Future studies may prospectively collect data from several centres to overcome low test power.

Within the limitations of the present study, the following conclusions regarding periodontal treatment of teeth with class II FI may be drawn:

– Periodontal treatment of teeth with class II FI in general results in a high rate of long‐term retention.
– Multiple class II FI at T1, RCF and premolars seem to deteriorate long‐term prognosis of teeth with class II FI.
– Regular SPC favours long‐term retention of teeth with class II FI.

## Author Contributions

P.E. and N.E.S. conceived and designed the study. T.C. collected the data. B.P. and B.D. supervised the methods. N.L. and T.C. analysed the data. M.S. managed the group. P.E. secured funding and led the writing. All authors contributed substantially to the interpretation of the data as well as to drafting and critically revising the manuscript. All gave their final approval of the version to be published and agreed to be accountable for all aspects of the work.

## Ethics Statement

All procedures performed in the study were approved by the Institutional Review Board for Human Studies of the Medical Faculty of Johann Wolfgang Goethe‐University Frankfurt am Main (approval number: 61/15) and conducted in accordance with the 1975 Declaration of Helsinki as revised in 2013.

## Conflicts of Interest

The authors declare no conflicts of interest.

## Supporting information


**Data S1.** Supporting information.

## Data Availability

The data that support the findings of this study are available from the corresponding author upon reasonable request.
