# Supplementary material for: Long‐Term Prognosis of Teeth With Class II Furcation Involvement: A Retrospective Cohort Study
Source: J Clin Periodontol. 2025 Jun 2;52(9):1298–305. doi: 10.1111/jcpe.14186 (PMC12377941; doi:10.1111/jcpe.14186)
Supplement: Supplementary file 1 — Data S1. Supporting information. [file JCPE-52-1298-s001.docx]

Long-term prognosis of teeth with class II furcation involvement: a retrospective cohort study

Eickholz P, Cordis T, Dannewitz B, Pretzl B, Schröder M, Lingwal N, El Sayed N

Supplement

Material and Methods

**Patients**

All charts of patients that had accomplished APT since October 2004 at the Center for Dentistry and Oral Medicine of Goethe-University Frankfurt/Main, Germany were screened for presence of teeth with class II FI. The study protocol resembles a recent study on long-term survival of teeth with class III FI (Eickholz et al., 2021). Patients had to fulfil the following criteria:

- Comprehensive periodontal treatment not prior to October 2004 consisting of oral hygiene instructions and supragingival debridement, followed by subgingival instrumentation (SI) based on a modified version (Eickholz et al., 2013) of the full-mouth disinfection (FMD) concept (Quirynen et al., 1995). The protocol for step 1 and 2 treatment is described in detail elsewhere (Eickholz et al., 2013). Re-evaluation of periodontal condition was performed 6 weeks to 3 month after SI and if required step 3 treatment performed (surgical treatment) (Sanz et al., 2020).
- At least one tooth with class II FI at least on one furcation entrance prior to start of treatment. A tooth was classified as class II FI if at least one furcation entrance exhibited class II FI. Further, in accordance with the 2020 EFP S3 clinical guideline we introduced the dichotomous variable “multiple class II FI” per tooth (yes/no) (Sanz et al., 2020).
- Complete periodontal charting with PPD and CAL-V at 6 sites/tooth, FI (Hamp et al., 1975) at all furcation entrances of multi-rooted teeth prior to treatment (baseline, T0) and after accomplishment of APT (re-evaluation 1 or 2 and start of SPC, T1). FI was exclusively assessed clinically using a Nabers probe (PQ2N, Hu-Friedy, Chicago, USA).
- Radiographs of all teeth at T0
- Follow-up ≥5 years after T1 (T2: complete periodontal charting ≥5 years after accomplishment of APT)
- SPC was conducted at the Department of Periodontology of Goethe-University by dentists in postgraduate periodontal training or board-certified periodontal specialists in collaboration with dental nurses or dental hygienists as well as by students under the respective dentists’ supervision. It was always structured identically (Petsos et al., 2020, Eickholz et al., 2008):

1. Modified GBI (Ainamo et al., 1975) and modified PCR (O'Leary et al., 1972)
2. Re-instruction and re-motivation for effective individual plaque control
3. Professional mechanical plaque removal with hand instruments and polishing by use of rotating rubber cups with polishing paste (SuperPolish, Kerr GmbH, Biberach, Germany)
4. Application of fluoride gel (Elmex Gelee, GABA Schweiz AG, Therwil, Switzerland)
5. Twice per year, recording of general dental and complete periodontal status including PPD, BOP, FI, and tooth mobility
6. Once per year scoring of CAL and sensitivity testing
7. At sites with PPD = 4 mm and BOP or PPD ≥5 mm SI and instillation of 1% chlorhexidine digluconate gel (Chlorhexamed 1% gel, GlaxoSmithKline GmbH, Munich, Germany)
8. Patients scoring a high periodontal risk according to the periodontal risk assessment (Eickholz et al., 2008) and therefore scheduled four times a year received complete SPT including the above-mentioned items 1-5 in 6 months intervals and SPC without dental and periodontal status (items 1-4) in between.
9. If a patient under SPC for at least 2 years exhibited > 5 teeth with PPD ≥ 5 mm a repetition of comprehensive periodontal therapy was recommended considering individual factors such as the patients’ age and/or the presence of systemic diseases.

**Analysis of patients‘ charts**

For each patient sex, age (T0) and time of follow-up (T1-T2) was extracted. For all teeth with class II FI that had been extracted, the time between T1 and the date of extraction was recorded. If the date of extraction could not be identified, the date of the last examination when the respective tooth was still present served as date of extraction for survival calculation. Based on baseline examination each patient was retrospectively assigned a diagnosis (e.g., periodontitis, generalized stage III, grade B) according to the 2018 classification (Papapanou et al., 2018). Total number of SPC visits and adherence to SPC was assessed for each patient. If a patient did not violate recommended SPC intervals by more than 100%, SPC was defined as regular. Violation of recommended intervals by more than 100% only once led to classification into irregular SPC (Eickholz et al., 2008). The following data were extracted from the patients‘ charts:

- Age at T0
- Smoking (active smoker, former smoker, non-smoker) (Lang and Tonetti, 2003) at T0,
- Diabetes mellitus (T0),
- Class of FI at all furcation entrances at T0, T1 and T2
- Root canal filling (RCF) at tooth with class II FI (T0),
- Treatment of teeth with class II FI:
  - SI, OFD
  - Systemic antibiotics adjunctive to SI
  - Resective treatment (root amputation, hemisection, trisection, root resection, tunnelling),
  - Type of roots resected
  - Regenerative treatment (barrier membranes or enamel matrix derivative with or without deproteinized bovine bone filler)
- Mean Plaque Control Record (PCR (O'Leary et al., 1972)) during SPC (T1 to T2)
- Most severe PPD and CAL per tooth with class II FI at T0 and T1.

**Analysis of radiographs**

Baseline radiographs (T0: panoramic or full sets of periapical intraoral radiographs) were viewed on a screen in a darkened room (T.C.). Type of bone loss was categorized into horizontal/vertical. Vertical bone loss (intrabony defects) was measured and categorized into shallow (≤ 3 mm) or deep (> 3 mm) intrabony defects using a scaled loupe (Peak Nr. 1983 Scale Lupe, 10x, Thokai Sangyo, Japan) (Eickholz et al., 1998).

Relative radiographic bone loss (distance cemento-enamel junction to alveolar crest in relation to root length) was assessed at the periodontally most affected site of each tooth with class II FI and at the tooth with most severe bone loss in the dentition (grade). Assessment was performed using a Schei ruler with 10% scale (Schei et al., 1959). The most coronal line of the Schei ruler was aligned perpendicularly to the tooth axis of the respective tooth and adjusted horizontally until its most apical line touched the root apex. Relative bone loss at the most severely affected interdental site of the respective tooth in relation to root length was assessed in 10% increments and always rounded to the next nearest 10%. Division of relative bone loss by patients’ age provided the bone loss age coefficient (Eickholz et al., 2020, Tonetti et al., 2018).

References

Eickholz, P., Kaltschmitt, J., Berbig, J., Reitmeir, P. & Pretzl, B. (2008) Tooth loss after active periodontal therapy. 1: patient-related factors for risk, prognosis, and quality of outcome. *J Clin Periodontol* **35,** 165-174. doi:10.1111/j.1600-051X.2007.01184.x.

Eickholz, P., Kim, T. S., Benn, D. K. & Staehle, H. J. (1998) Validity of radiographic measurement of interproximal bone loss. *Oral Surg Oral Med Oral Pathol Oral Radiol Endod* **85,** 99-106. doi:10.1016/s1079-2104(98)90406-1.

Eickholz, P., Runschke, M., Dannewitz, B., Nickles, K., Petsos, H., Kronsteiner, D. & Pretzl, B. (2021) Long-term prognosis of teeth with class III furcation involvement. *J Clin Periodontol* **48,** 1528-1536. doi:10.1111/jcpe.13551.

Eickholz, P., Schroder, M., Asendorf, A., Schacher, B., Oremek, G. M., Kaiser, F., Wohlfeil, M. & Nibali, L. (2020) Effect of nonsurgical periodontal therapy on haematological parameters in grades B and C periodontitis: an exploratory analysis. *Clin Oral Investig*. doi:10.1007/s00784-020-03292-7.

Eickholz, P., Siegelin, Y., Scharf, S., Schacher, B., Oremek, G. M., Sauer-Eppel, H., Schubert, R. & Wohlfeil, M. (2013) Non-surgical periodontal therapy decreases serum elastase levels in aggressive but not in chronic periodontitis. *J Clin Periodontol* **40,** 327-333. doi:10.1111/jcpe.12076.

Lang, N. P. & Tonetti, M. S. (2003) Periodontal risk assessment (PRA) for patients in supportive periodontal therapy (SPT). *Oral Health Prev Dent* **1,** 7-16.

O'Leary, T. J., Drake, R. B. & Naylor, J. E. (1972) The plaque control record. *J Periodontol* **43,** 38. doi:10.1902/jop.1972.43.1.38.

Papapanou, P. N., Sanz, M., Buduneli, N., Dietrich, T., Feres, M., Fine, D. H., Flemmig, T. F., Garcia, R., Giannobile, W. V., Graziani, F., Greenwell, H., Herrera, D., Kao, R. T., Kebschull, M., Kinane, D. F., Kirkwood, K. L., Kocher, T., Kornman, K. S., Kumar, P. S., Loos, B. G., Machtei, E., Meng, H., Mombelli, A., Needleman, I., Offenbacher, S., Seymour, G. J., Teles, R. & Tonetti, M. S. (2018) Periodontitis: Consensus report of workgroup 2 of the 2017 World Workshop on the Classification of Periodontal and Peri-Implant Diseases and Conditions. *J Clin Periodontol* **45 Suppl 20,** S162-S170. doi:10.1111/jcpe.12946.

Petsos, H., Schacher, B., Ramich, T., Nickles, K., Dannewitz, B., Arendt, S., Seidel, K. & Eickholz, P. (2020) Retrospectively analysed tooth loss in periodontally compromised patients: Long-term results 10 years after active periodontal therapy-Patient-related outcomes. *J Periodontal Res* **55,** 946-958. doi:10.1111/jre.12786.

Quirynen, M., Bollen, C. M. L., Vandekerckhove, B. N. A., Dekeyser, C., Papaioannou, W. & Eyssen, H. (1995) Full- vs. Partial-mouth Disinfection in the Treatment of Periodontal Infections: Short-term Clinical and Microbiological Observations. *Journal of Dental Research* **74,** 1459-1467. doi:10.1177/00220345950740080501.

Sanz, M., Herrera, D., Kebschull, M., Chapple, I., Jepsen, S., Beglundh, T., Sculean, A., Tonetti, M. S., Participants, E. F. P. W. & Methodological, C. (2020) Treatment of stage I-III periodontitis-The EFP S3 level clinical practice guideline. *J Clin Periodontol* **47 Suppl 22,** 4-60. doi:10.1111/jcpe.13290.

Schei, O., Waerhaug, J., Lovdal, A. & Arno, A. (1959) Alveolar Bone Loss as Related to Oral Hygiene and Age. *Journal of periodontology* **30,** 7-16.

Tonetti, M. S., Greenwell, H. & Kornman, K. S. (2018) Staging and grading of periodontitis: Framework and proposal of a new classification and case definition. *J Clin Periodontol* **45 Suppl 20,** S149-S161. doi:10.1111/jcpe.12945.
